# Metastatic lobular carcinoma of breast mimics primary cervix carcinoma: two case reports and a review of the literature

**DOI:** 10.3332/ecancer.2015.571

**Published:** 2015-09-10

**Authors:** Rajitha Lokadasan, K Ratheesan, Renu Sukumaran, Sindhu P Nair

**Affiliations:** 1 Department of Medical Oncology, Regional Cancer Centre, Medical College Campus, Post Bag No 2417, Trivandrum 695011, India; 2 Department of Radiotherapy, Regional Cancer Centre, Trivandrum 695011, India; 3 Department of Pathology, Regional Cancer Centre, Trivandrum 695011, India

**Keywords:** lobular carcinoma, breast, metastatic, female genital tract, cervix, endometrium

## Abstract

Invasive lobular carcinoma (ILC) of the breast exhibits unusual clinicopathological, radiological, histological, and metastatic patterns. We present here two cases of ILC of the breast that presented with an unusual pattern of metastasis involving the uterus.

Our first patient presented to her primary gynaecologist with profuse vaginal bleeding and underwent total abdominal hysterectomy and bilateral salpingo-oophrectomy. She had fibroadenoma excised from her left breast four years previously. Histopathology revealed lobular carcinoma diffusely infiltrating uterus, cervix, and bilateral ovaries. Retrospective examination of the left breast showed induration along the previous fibroadenoma excision scar. A biopsy from the scar suggested lobular carcinoma.

Our second patient presented with a hard indurated cervix mass that mimicked primary cervix carcinoma. She had ILC of the right breast four years previously for which she underwent mastectomy followed by adjuvant chemotherapy and radiotherapy. She was on tamoxifen. Further evaluation at presentation with imaging showed extensive intra-abdominal disease involving peritoneum with moderate ascites, adnexal masses, and confluent para-aortic nodal mass. A cervix biopsy confirmed metastasis from lobular carcinoma.

Metastatic involvement of the genital tract should be considered in women with a history of breast cancer who present with abnormal vaginal bleeding, suspicious pelvic examination, or radiological findings. We suggest such patient be vigorously screened with biopsy even if the patient is disease-free for several years. It is crucial to differentiate the metastasis from primary carcinoma of the genital tract as there are vast differences in the management of each.

## Introduction

Metastatic lobular carcinoma of the breast tends to exhibit unusual sites of metastasis. We present two case reports of lobular carcinoma of the breast presenting with metastasis to the uterus and cervix. A high index of clinical suspicion, optimal imaging modalities, and detailed histopathologic examination is required for the correct diagnosis. Correct diagnosis is imperative as the management for primary genital tract carcinoma and metastatic lobular carcinoma are dramatically different.

## Case reports

### Patient 1

**Figure 1.**
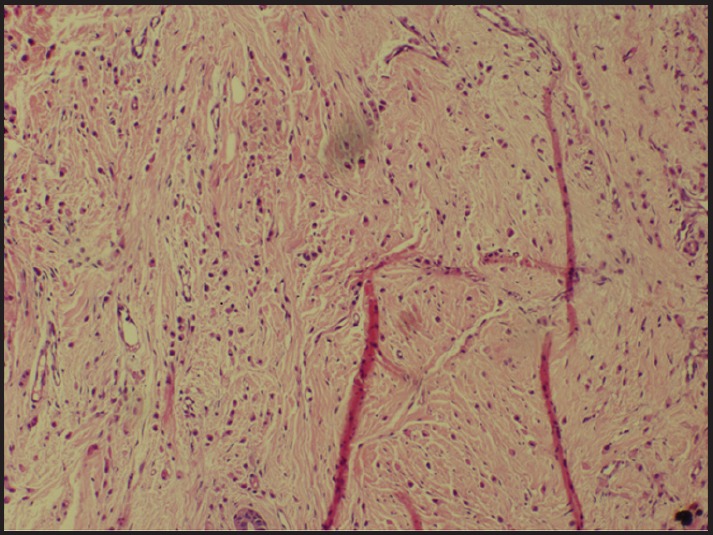
Tumour cells infiltrating myometrium in single-file linear or Indian file pattern (H & E × 100).

A 49-year-old pre-menopausal lady presented to her primary gynaecologist with menorrhagia of six months duration. At age 45 she had a left breast lump excision biopsy which was reported as a fibroadenoma. Clinical examination revealed a bulky cervix and uterus. A transvaginal ultrasound scan showed only fibroid uterus with endometrial polyp and minimal free fluid in the pouch of Douglas. At presentation, she had refractory bleeding requiring multiple blood transfusions. She was then taken up for prophylactic total abdominal hysterectomy and bilateral salpingo-oophrectomy. Histopathological examination revealed metastatic deposits of a lobular pattern diffusely involving the endometrium, myometrium, fibroid, cervix, and bilateral ovaries (Figure 1). The possibility of metastasis from lobular carcinoma of breast was considered. A right breast mammogram was BIRADS category 1 with no abnormal lesions. The left breast could not be mammographied because of scanty breast tissue. She presented to us with a diagnosis of metastatic lobular carcinoma involving genital tract and primary could not be detected. However, careful palpation of the scar in the left breast showed induration and two left axillary lymph nodes of size 1 × 1 cm. Fine needle aspiration from the scar revealed epithelial proliferation with cytological atypia.

**Figure 2.**
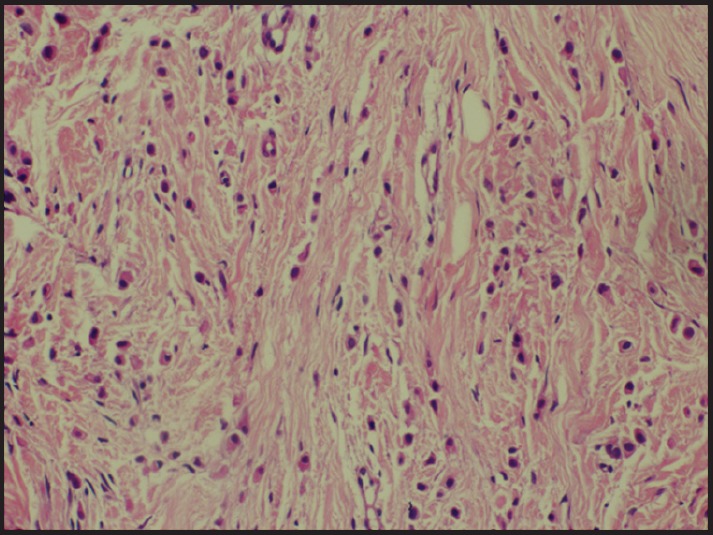
Tumour cells loosely dispersed throughout fibrous matrix (H & E × 200).

**Figure 3.**
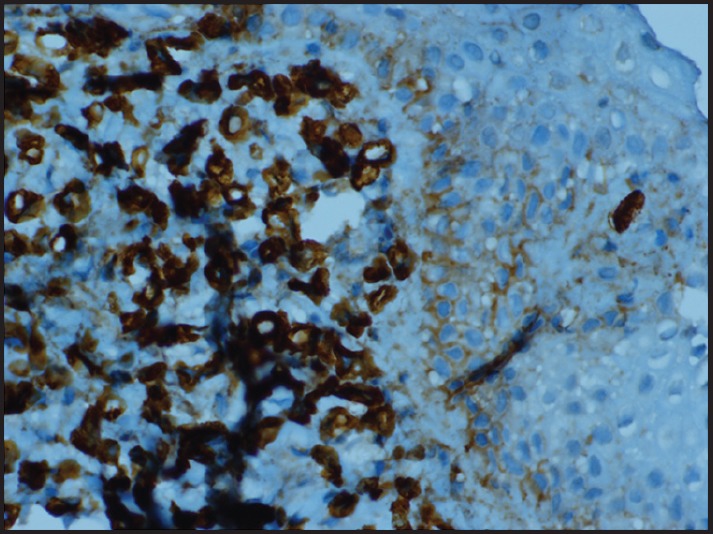
Tumour cells showing CK7 positivity.

Following this, a biopsy from the scar over the left breast was taken that showed ILC positive for cytokeratin and gross cystic disease fluid protein (GCDFP) and negative for E cadherin (Figure 2, 3). Oestrogen receptor (ER) and progesterone receptor (PR) were diffuse to weak positive and HER-2 negative. Postoperative CT chest abdomen and pelvis showed no metastasis. Bone scan was normal. She was started on palliative chemo with 5-fluorouracil, epirubicin, cyclophosphamide following which hormone therapy was planned.

### Patient 2

A 49-year-old woman presented with abdominal distension and pain. She did not have abnormal vaginal bleeding or discharge. Four years previously she had a left breast lump biopsy which suggested ILC grade II with *in situ* component. She was cT3N2M0. Tumour was weak positive for ER and diffuse strong positive for PR. She underwent left modified radical mastectomy. Postoperative histology showed no evidence of residual tumour and 11 out of 16 lymph nodes dissected were positive. Subsequently, she received adjuvant chemotherapy with three cycles of 5-fluorouracil, adriamycin, cyclophosphamide followed by three cycles of docetaxel. Following this, adjuvant radiotherapy was administered to chest wall and drainage areas at a dose of 50 Gy in 25 fractions. Simultaneously, she was started on tamoxifen. She was on regular follow-up from the breast clinic without any evidence of recurrence.

Physical examination at the time of admission showed Eastern Cooperative Oncology Group (ECOG) performance status 2 with bilateral pedal oedema. There was no pallor or lymph node enlargement. There was no evidence of local recurrence. The opposite breast and axilla were normal. An abdominal wall oedema was elicited with no other significant findings. Pelvic examination disclosed a hard mass of cervix obliterating the fornices and of restricted mobility. The first possibility of a second malignancy of cervix was considered clinically.

**Figure 4.**
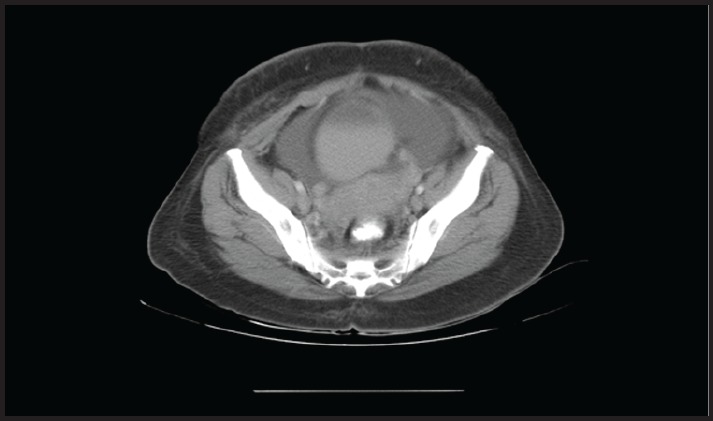
CT image showing adnexal mass.

Further investigation with CT abdomen and pelvis showed moderate to gross ascites with irregular soft tissue stranding in the omentum. Bilateral irregular soft tissue density mass lesions noted in adnexa, on the right measuring 42 × 33 mm and on the left measuring 43 × 21 mm. (Figure 4). Para-aortic nodal mass seen as heterogenous conglomerate soft tissue attenuation. The stomach showed diffuse irregular wall thickening.

**Figure 5.**
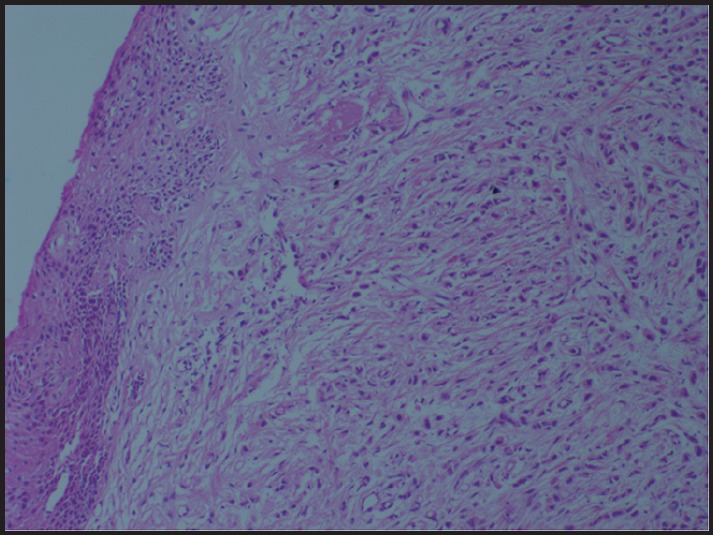
Section showing tumour cells infiltrating cervix H & E × 100.

Endoscopic-guided biopsy of the stomach was done which was negative for malignancy. Ascitic fluid cytology revealed adenocarcinoma. A punch biopsy from the cervix mass showed poorly differentiated adenocarcinoma with lobular growth pattern and occasional signet ring cells. (Figure 5). The metastatic tumour cells were strongly positive for cytokeratin and negative for E cadherin. Tumour cells exhibited immunopositivity for ER and PR similar to the initial tumour.

The final diagnosis was lobular carcinoma of the breast presenting with extensive metastases as bilateral adnexal masses, omental deposits, and hard cervix mass. She was started on second line chemotherapy with carboplatin and gemcitabine.

## Discussion

Metastases in the female genital tract from an extragenital primary tumour is uncommon. Mazur *et al* in 1984 analysed 325 cases of metastasis to female genital tract. [1] The most frequent metastatic sites for extragenital primaries were ovaries followed by vagina. Despite trends in distribution of metastasis, it was demonstrated that all sites in the female genital tract are at risk for metastasis. The majority of the extragenital primaries metastasing to gynaecological organs were adenocarcinoma from gastrointestinal tract. They originated less frequently from the breast and other organs.

Malignancies metastatic to ovaries account for 15% of all ovarian malignancies [2]. The most common sites of primary were from the colon, endometrium, breast, appendix, and stomach [3]. The metastases from the breast, stomach, and appendix carcinoma were found to be often bilateral. Differentiating a primary ovary carcinoma from metastatic ovary carcinoma and detection of the primary prior to surgery is important and has favourable prognostic impact. Metastasis from the colon has the worst prognosis as compared to other nongenital sites [4]. Immunohistochemistry is often helpful to identify the primary site [5].

The most common source of metastatic involvement of the uterus is carcinoma of the breast. Kumar *et al* published a review in 1988 with 63 cases of metastatic cancers to uterine corpus from extragenital neoplasms [6]. The most common primary metastasising to uterus in this series was breast cancer (47.3%). Interestingly, the myometrium was found to be more involved than the endometrium although most of the endometrial curettage showed metastatic tumour cells.

Metastasis of non-gynaecologic tumours to the cervix is a rare event. Of 325 metastatic female genital neoplasms, Mazur *et al* found only 3.7% involved the cervix, and none of these represented primary breast carcinomas [1]. The cervix being a small target organ constituted predominantly of fibromuscular tissue with limited blood supply and only afferent lymphatic drainage making it a less favourable site for metastasis. Pérez-Montiel D *et al* reports the most common tumour metastasing to cervix are gastrointestinal tract and ovarian cancers [7]. Breast cancer metastasising to cervix uteri is very rare. Only a few cases have been reported so far. Considering the rarity the actual incidence of cervix metastasis is unknown.

Metastatic progression of breast cancer is highly unpredictable and depends on the inter-tumoural and intra-tumoural heterogeneity and the phenotypic evolution during disease progression. The autopsy findings in 197 females who died of metastatic breast cancer by Cumming *et al* showed that younger females are at higher risk for metastasis to gynaecological sites [8]. This is probably because in younger females the oestrogen-rich environment of pre-menopausal ovary provides a favourable site for metastasis.

Lobular histotype seems to metastasise to the genital tract more frequently than ductal tumours [9]. The incidence of ILC metastasising to the genital tract is 36%–52% in autopsy series and 2%–5% in clinical series. Le Bouedec *et al* in 1993 described 12 patients with metastatic breast cancer to the uterus out of which ten had ILC [10].

Unlike invasive ductal carcinoma, lobular carcinoma exhibits a distinct metastatic pattern. Invasive ductal carcinoma tends to metastasise more commonly to the liver, lung, and brain while lobular carcinoma metastasise more to bones, peritoneum, retroperitoneum, gastrointestinal tract, and genitourinary tract [11]. Contralateral breast carcinomas are more common with lobular carcinoma than ductal carcinomas. There are several case reports and series that report unusual sites of metastasis for lobular carcinoma of breast.

The exact mechanism for this unusual metastatic pattern is not known. Loss of E cadherin expression on tumour cell membrane is a characteristic feature of ILC. This loss results from inactivation of CDH1 gene at 16q22. Loss of E cadherin expression is associated with abnormalities in catenin expression leading to loss of cell-to-cell adhesion and intracellular and intercellular signalling. This is postulated as the mechanism for the unusual metastatic pattern of ILC [12]. Knowledge regarding the unusual metastatic pattern of lobular carcinoma is essential to differentiate it from second primary and then plan appropriate treatment.

In the literature most of the cases of breast cancer with genital tract metastasis occurred in advanced breast cancer while on tamoxifen treatment or during follow-up. There are only a few cases where genital tract involvement as initial presentation was reported [13]. This highlights the rarity of presentation in our first case. The patient underwent hysterectomy for vaginal bleeding and the breast cancer was diagnosed later. Our second patient presented with extensive peritoneal metastasis and hard mass of cervix four years following the diagnosis of the primary tumour. C A Bryson *et al* reviewed 27 cases of breast carcinoma metastasising to the cervix. In most reports there were multiple metastases presenting on an average interval of 44.5 months between primary and secondary diagnosis as in our patient [14].

Radiologically lobular carcinomas are more difficult to detect with mammogram. This is probably because of the fact that lobular tumours tend to grow as sheets of cancer cells rather than discrete masses and induce less desmoplastic reaction. Hence ILC often fails to form a distinct mass in the breast. This accounts for lesser radiographic density and delayed detection in mammography. Similar diffuse spreading process is also described at the metastatic sites [15]. Harris *et al* reports the diffuse infiltration of metastatic ILC breast in the stomach that resembles linitis plastica and make it indistinguishable from primary scirrhous carcinoma [16], Likewise in our first patient, initial imaging failed to reveal the metastatic lesions in the uterus and cervix. Subsequent histopathology only confirmed the diagnosis. However, the second patient had hard nodular cervix mass, bilateral adnexal masses, and confluent para-aortic nodes probably because of the advanced nature of the illness. The radiology mimicked a more common differential diagnosis like primary metastatic ovary carcinoma rather than metastatic lobular breast carcinoma. In the case series by Yazigi *et al*, out of 27 cases summarised more than 60% had no evidence of disease on examination and the metastasis would have been missed if complete evaluations had not been performed. [17] Taking into consideration the case series and as in our case, we see it is easy to mistake the common presentation of abnormal vaginal bleeding as primary disease rather than metastatic involvement of genital tract.

It is imperative to differentiate the metastasis from primary carcinoma of genital tract as the management is dramatically different. The histological diagnosis of the secondary deposit is crucial for the correct diagnosis. Microscopically, metastases from ILC consist of spindle-shaped cells usually that show a single-file growth pattern with no dominant mass. The most important markers for ILC are GCDFP-15, loss of E Cadherin, positive for ER and PR. The histological recognition of lobular carcinoma at the metastatic sites may be difficult. Nevertheless, in our two cases the metastatic sites maintained their characteristics to the primary. Studies have found ILC more likely to be ER+/PR+ than ductal carcinomas. HER-2 protein overexpression or gene amplification have been reported in <1% to 6.2% of all ILC cases. Few ILC cases (1.5%) exhibit a triple negative phenotype [12]. Both of our patients were positive for ER and PR and negative for HER-2 protein . In the first case tumour cells had signet ring morphology. Signet ring cell carcinoma of the breast is usually considered to be a morphologic variant of infiltrating lobular carcinoma, with a poor prognosis [16].

An autopsy series by Harris *et al* described further differences in the metastatic pattern in addition to the sites involved between invasive lobular and ductal carcinoma [16]. The metastatic pattern in ILC was diffuse or as tiny nodules of 1 to 2 mm size and it tends to become confluent in heavily infiltrated regions, while invasive ductal carcinoma (IDC) involvement was more nodular masses rather than diffuse. Our first patient showed similar diffuse infiltrating disease involving bilateral ovaries, uterus, and cervix without discrete masses. The advanced nature of the illness can probably explain the confluent masses seen in the second patient. Moreover, the inflammatory cellular response and fibrous proliferation of cervix to metastatic disease may explain the hard indurated cervix mass in our patient.

ILC constitutes only 5%–15% of invasive breast carcinoma. Because of the scarcity of this entity, evidence regarding treatment outcome and prognosis is lacking. Conflicting reports have been reported regarding prognosis of ILC as compared to IDC [12]. A large series of 12,506 breast cancer patients entered in 15 International Breast Cancer Study Group (IBCSG) trials demonstrated the prognosis for lobular carcinoma to be better than for ductal carcinoma in the early years. However, the relapse rate progressively increased and surpassed ductal carcinoma at six years. This is postulated to be because of the ER positive lobular carcinoma tends to relapse late. The median survival of individuals with metastatic breast cancer is 18–24 months [18]. In the retrospective analysis by Sanuki-Fujimoto N *et al* prognosis of patients with unusual metastases was similar to that of patients with metastasis only at usual sites [19].

## Conclusion

In the two case reports presented, the female genital tract is the host of infiltrating lobular carcinoma of the breast. The diagnosis of genital metastases secondary to breast cancer can be difficult for several reasons. The reasons postulated are nonspecific symptoms at presentation or abnormal vaginal bleeding, long disease-free interval, challenging radiology, and histopathology. Hence diagnosis requires a high index of clinical suspicion. We suggest any woman with breast cancer presenting with gynaecologic symptoms should be screened for metastatic disease including a biopsy. Accurate diagnosis is essential as the treatments for primary genital tract carcinoma and metastatic lobular carcinoma are vastly different.

## Grant support

Not applicable.

## Financial disclosures

All authors have read the manuscript and claim that there is no conflict of interest or financial disclosure.

## References

[1] Mazur MT, Hsueh S, Gersell DJ (1984). Metastases to the female genital tract. Analysis of 325 cases. Cancer.

[2] De Waal YR (2009). Secondary ovarian malignancies: frequency, origin, and characteristics. Int J Gynecol Cancer.

[3] Bruls J (2015). A national population-based study provides insight in the origin of malignancies metastatic to the ovary. Virchows Arch.

[4] Skírnisdóttir I, Garmo H, Holmberg L (2007). Non-genital tract metastases to the ovaries presented as ovarian tumors in Sweden 1990–2003: occurrence, origin and survival compared to ovarian cancer. Gynecol Oncol.

[5] Kondi-Pafiti A (2011). Metastatic neoplasms of the ovaries: a clinicopathological study of 97 cases. Arch Gynecol Obstet.

[6] Kumar NB, Hart WR (1982). Metastases to the uterine corpus from extragenital cancers. A clinicopathologic study of 63 cases. Cancer.

[7] Pérez-Montiel D (2012). Adenocarcinoma metastatic to the uterine cervix: a case series. J Obstet Gynaecol Res.

[8] Cummings MC (2014). Metastatic progression of breast cancer: insights from 50 years of autopsies. J Pathol.

[9] Hongying He (2014). Distant Metastatic Disease Manifestations in Infiltrating Lobular Carcinoma of the Breast. AJR Am J Roentgenol.

[10] Le Bouedec G (1993). Arch AnatCytolPathol. Uterine metastases originating from breast cancer. Apropos of 12 cases. Arch Anat Cytol Pathol.

[11] Cao AY (2012). Tumor characteristics and the clinical outcome of invasive lobular carcinoma compared to infiltrating ductal carcinoma in a Chinese population. World J Surg Oncol.

[12] Séverine Guia, Anita Wolfer B (2014). Invasive lobular breast cancer and its variants: How special are they for systemic therapy decisions?. Crit Rev Oncol Hematol.

[13] Engelstaedter V, Mylonas I (2011). Lower genital tract metastases at time of first diagnosis of mammary invasive lobular carcinoma. Arch Gynecol Obstet.

[14] Bryson CA, de Courcy-Wheeler RH (1999). Breast cancer metastasising to the uterine cervix. Ulster Med J May.

[15] Winston CB (2000). Metastatic lobular carcinoma of the breast: patterns of spread in the chest, abdomen, and pelvis on CT. Am J Roentgenol.

[16] Harris M (1984). A comparison of the metastatic pattern of infiltrating lobular carcinoma and infiltrating duct carcinoma of the breast. Br Cancer.

[17] Roberto Yazigi MD (1988). Breast cancer metastasizing to the uterine cervix. Cancer.

[18] Pestalozzi BC, Zahrieh D, Mallon E (2008). Distinct clinical and prognostic features of infiltrating lobular carcinoma of the breast: combined results of 15 International Breast Cancer Study Group clinical trials. J Clinical Oncology.

[19] Sanuki-fujimoto N (2008). Pattern of tumor recurrence in initially nonmetastatic breast cancer patients: distribution and frequency of metastases at unusual sites. Cancer.

